# Meningeal metastatic tumor with bone destruction from follicular thyroid carcinoma: a case report and literature review

**DOI:** 10.3389/fsurg.2025.1708113

**Published:** 2026-01-06

**Authors:** Wei Liu, Lanming Su, Qinglu Zhang, Yuanqin Liu

**Affiliations:** 1 Department of Neurosurgery, The First Affiliated Hospital of Shandong First Medical University and Shandong Provincial Qianfoshan Hospital, Jinan, China; 2 Department of Neurology, The Second Affiliated Hospital of Shandong First Medical University, Taian, Shandong, China; 3 Department of Ultrasound Medicine, Shandong Provincial Third Hospital, Shandong University, Jinan, Shandong, China

**Keywords:** bone destruction, follicular thyroid carcinoma, intracranial metastasis, meningeal metastasis, meningioma mimicry

## Abstract

**Background:**

Follicular thyroid carcinoma (ftc) is a malignant neoplasm arising from thyroid follicular epithelial cells and is the second most common thyroid cancer after papillary thyroid carcinoma. meningeal metastasis from ftc with calvarial destruction is exceedingly rare and is often misdiagnosed clinically as meningioma. we reviewed previously reported cases in the literature and, in addition, report and include one case of meningeal metastasis from ftc treated at our center.

**Methods:**

Using the keywords “meningioma,” “follicular thyroid carcinoma,” and “cerebral metastatic tumor,” we searched PubMed/MEDLINE and Web of Science to identify relevant publications. We extracted patient age, sex, tumor location and size, treatment modalities, and follow-up outcomes. In parallel, we provide a detailed description and analysis of the clinical course of the present case.

**Results:**

A total of 10 patients were included, comprising the cases reported in the literature and the index case in this study. The mean age was 60.5 years, and most lesions were solitary epidural or dural-based masses. Headache was the most common presenting symptom. All patients underwent surgical resection; postoperative recurrence occurred in two cases, and the longest survival on follow-up was 7 years.

**Conclusions:**

Meningeal metastasis from FTC is rare and typically presents as a dural-based mass that readily mimics meningioma. Gross-total resection can achieve favorable initial disease control, although recurrence may still occur. Given the small number of reported cases, the long-term prognosis remains uncertain and warrants further investigation.

## Introduction

Thyroid cancer is the most common endocrine malignancy. Based on cellular origin, it is classified into tumors arising from follicular cells and from parafollicular cells (medullary carcinoma). Neoplasms derived from follicular cells account for 95% of all thyroid cancers (1) and are further subdivided into papillary thyroid carcinoma (PTC), follicular thyroid carcinoma (FTC), and anaplastic (undifferentiated) carcinoma (ATC). According to current international standards, including the 2015 American Thyroid Association (ATA) management guidelines and the European Society for Medical Oncology (ESMO) guidelines, the standard diagnostic work-up for thyroid nodules comprises high-resolution ultrasonography and fine-needle aspiration (FNA) cytology to assess the risk of malignancy (2, 3). Therapeutically, surgery—total thyroidectomy or lobectomy—remains the mainstay for differentiated thyroid cancer (DTC); in high-risk patients, postoperative radioactive iodine (RAI) therapy and thyroid-stimulating hormone (TSH) suppression are commonly employed to minimize recurrence and to treat distant metastasis (2). Among all histologic subtypes, FTC ranks second to PTC in incidence and may spread hematogenously or via lymphatics to lymph nodes, lungs, bone, liver, and other organs; however, meningeal metastasis presenting as a meningioma is rarely reported. Here, we describe a rare case of FTC with meningeal metastasis and calvarial destruction, and we conduct a systematic review of previously published cases to summarize the clinical characteristics and management of this entity.

## Methods

We included English-language studies published between 2002 and 2024 that were indexed in PubMed/MEDLINE and Web of Science. Searches used the keywords “meningioma,” “follicular thyroid carcinoma,” and “brain metastasis.” Sixteen records were initially identified and underwent detailed screening. We excluded reports of meningiomas metastasized from other adenocarcinoma types (e.g., breast, prostate) and required histopathological and immunohistochemical confirmation of a follicular thyroid carcinoma origin for inclusion. Ultimately, six studies met the criteria. From each, we extracted data on age at presentation, sex, symptoms, number and size of lesions, immunohistochemical findings, treatment strategies, and follow-up outcomes (Table 1). In addition, we collected and analyzed one case from our institution of meningeal metastasis arising from follicular thyroid carcinoma, confirmed intraoperatively and by pathology, and incorporated it into the overall case series.

**Table 1:** The abstracts of all confirmed cases of follicular thyroid carcinoma with leptomeningeal metastasis in the literature, including the current case study.

Author (Year)	Age	Gender (F/M)	Presenting symptom	Tumor location	Convexity meningioma manifestations (YES/NO)	Associated bone destruction (YES/NO)	Solitary (YES/NO)	Tumor size (cm)	Total resection	Follow-up results
Patricio Tagle(2002) (4)	50	F	Asymptomatic	Left temporal lobe	YES	NO	YES	Not reported	YES	Recurrence at the skull base was detected in the seventh year.
J. Ehrmann (2004) (5)	60	M	Headache and slurred speech	Cerebellopontine angle (CPA)	NO	NO	YES	6.2 × 6.5 × 5	YES	Not reported
Lesly Portocarrero-Ortiz(2009) (6)	61	F	Headache	Left temporoparietal region	YES	NO	YES	Not reported	YES	Not reported
Hamzaini Abdul Hamid (2009) (7)	43	F	Headache, blurred vision, and weakness in the left leg	Parafalcine	NO	NO	YES	5.1 × 4.6	YES	Not reported
Jun Shen (2015) (8)	67	M	Blurred vision	Right frontal lobe	YES	YES	YES	2.8 × 3.4 × 3.5	YES	Death occurred 22 months later due to widespread metastasis
	61	F	Asymptomatic	Parieto-occipital region	YES	YES	YES	8.0 × 8.0	YES	Not reported
	67	M	Local ulceration with bleeding	Occipital region	YES	YES	YES	3 × 3	YES	No recurrence for 4 years.
Marcus Unterrainer (2017) (9)	72	F	Headache and dizziness	Multiple occurrences	YES	NO	NO	Not reported	Not reported	Not reported
Abdulkerim Gokoglu (2023) (10)	52	F	Headache, dizziness, and numbness in all four limbs	Juxtasagittal	YES	YES	YES	4.1 × 5.3	Yes	No evidence of recurrence was observed during the 5-year follow-up period
Wei Liu (2024)	72	F	Headache, dizziness, and numbness of the scalp	Left frontotemporal region	YES	YES	YES	3 × 3	YES	No recurrence was detected in four years.

## Results

### Case series overview

Following a systematic search and screening, we included seven publications; together with our index case, the series comprised 10 patients (Table 1). At initial diagnosis, the mean age was 60.5 years (SD 9.94; range 43–72), indicating a predominance of middle-to-older adults, with no adolescent-onset cases identified. Females accounted for 70% (7/10) and males for 30% (3/10). Most patients presented with headache and a progressively enlarging cranial mass. A minority reported dizziness, limb weakness, blurred vision, or dysarthria. Symptomatology was concordant with lesion location, consistent with mass effect and/or injury to adjacent eloquent neural structures. In 8 of the 10 cases (80%), the metastases presented radiologically as convexity meningioma-like lesions, and skull erosion was documented in only two patients. Only one patient had multiple meningeal metastases; the remaining nine had solitary lesions. Reflecting the greater operability of solitary disease, no follow-up was curtailed by early postoperative mortality. The longest documented follow-up was 7 years, at which time recurrence had occurred.

### Detailed description of the index case

A 71-year-old woman presented with a >4-month history of a left temporal mass (Table 2). She had incidentally noticed a 3 × 3 cm lesion over the left temporal region protruding beneath the scalp, accompanied by intermittent headache, scalp paresthesia, and mild dizziness. She denied nausea, vomiting, blurred vision, visual field deficits, dysarthria, or limb motor impairment. Since symptom onset, mood and sleep quality had been poor. She reported a “thyroid operation” performed locally more than 10 years earlier, with unknown indication and procedure. Otherwise, she was in good health, with no history of diabetes, hypertension, or coronary artery disease.

**Table 2:** Timeline of clinical events of the present case.

Time point	Clinical Events and Interventions
>10 years ago	The patient underwent thyroid surgery in the local area (specific pathology unknown)
4 months prior to admission	Incident discovery of a 3 cm × 3 cm mass in the left temporal region protruding from the scalp
From 4 months prior to admission	Onset of intermittent headaches, scalp numbness, and mild dizziness; poor sleep and mental well-being
Admission	CT examination revealed a 4.8 cm × 4.2 cm lesion in the left frontotemporal bone with bone destruction and calcification
Hospitalization (Surgery)	Underwent “left temporal mass resection” under general anesthesia. Intraoperative findings showed a red tumor with rich blood supply adherent to muscles; blood transfusion was required
Post-operative (Pathology)	Histopathology and immunohistochemistry (CK+, TTF-1+, Tg+) confirmed the diagnosis of Follicular Thyroid Carcinoma
Post-operative (Adjuvant Therapy)	The patient received comprehensive treatment, including I131 therapy
Follow-up	Short-term follow-up indicated good recovery with no abnormal discomfort

**Figure 1:**
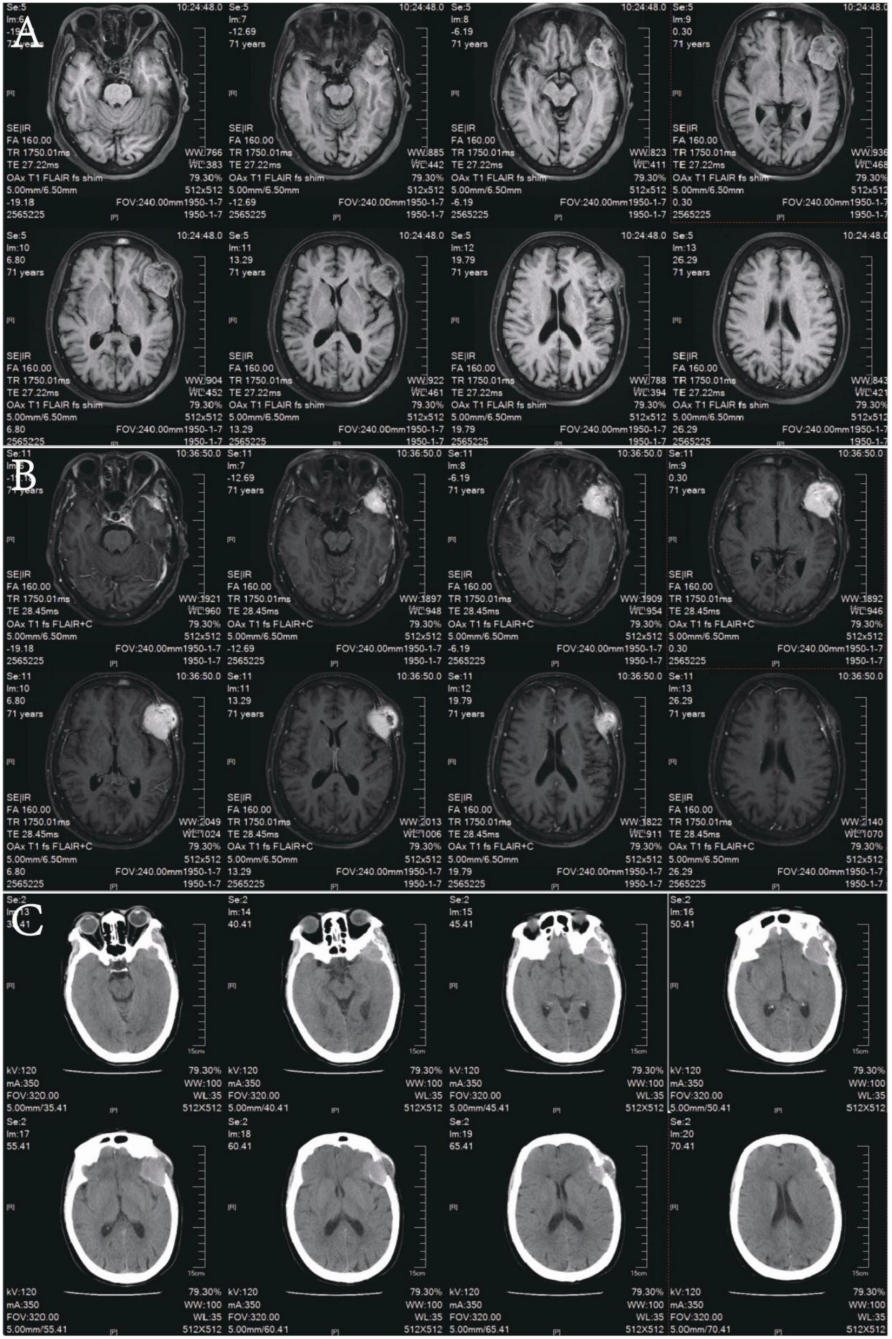
Pre-operative imaging of the patient. **(A)** T1-weighted MRI. **(B)** Contrast-enhanced MRI. **(C)** CT scan.

**Figure 2:**
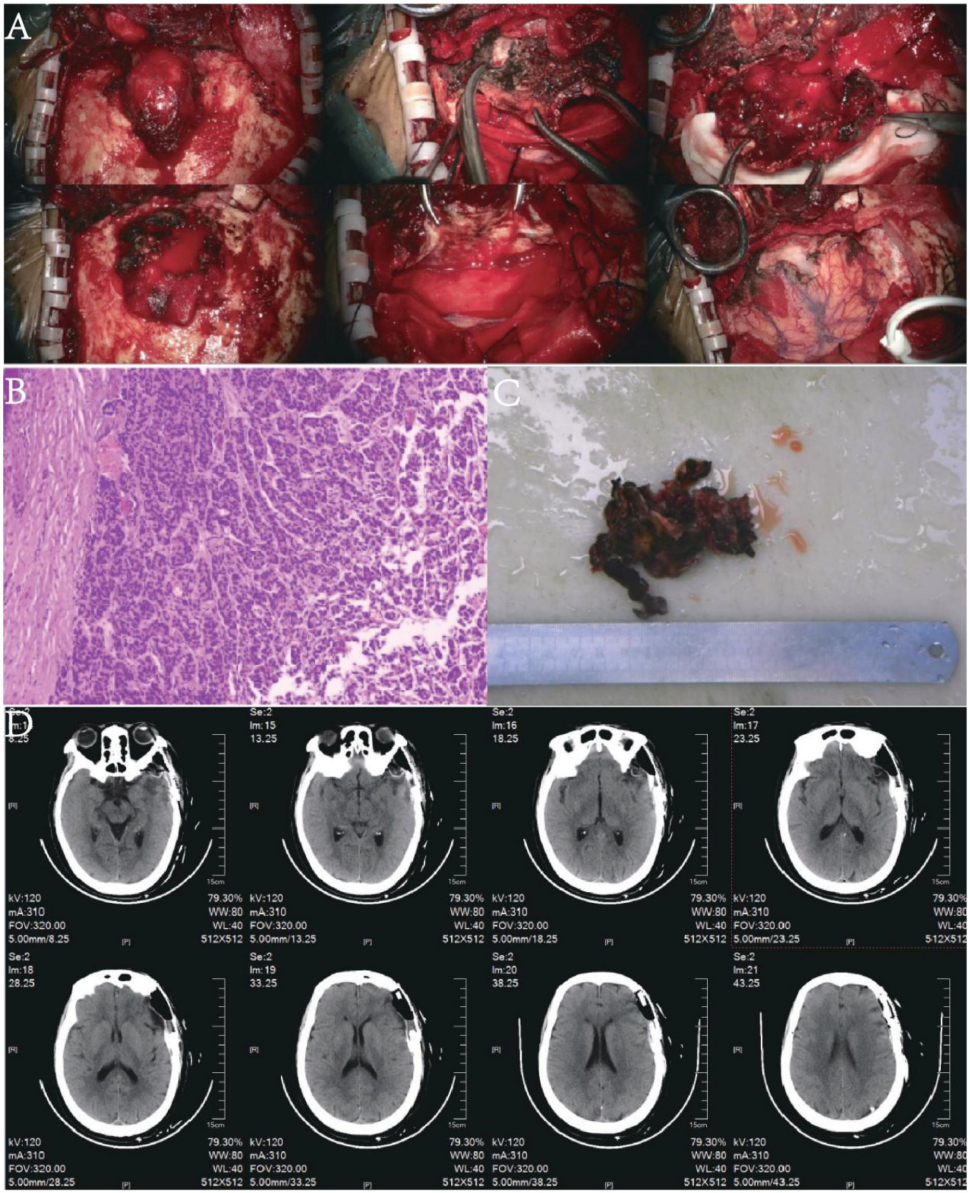
Intraoperative and postoperative imaging; **(A)** intraoperative photographs, from left to right: tumor exposure to complete resection; **(B)** histopathological staining of tumor tissue; **(C)** resected tumor specimen; **(D)** postoperative CT scan.

Head CT on admission (Figure 1C) revealed a quasi-round isodense lesion involving the inner and outer tables of the left frontotemporal calvarium, measuring approximately 4.8 × 4.2 cm in cross-section. Punctate calcifications were noted along the lesion margin; adjacent brain parenchyma showed mass effect with mild sulcal effacement. The neighboring calvarium exhibited osteolysis with irregular, discontinuous bone contours. MRI (Figures 1A,B) demonstrated a quasi-round lesion located on both the inner and outer tables of the left frontotemporal bone, showing iso- to slightly hyperintense signals on T1-weighted imaging, iso- to mildly hyperintense signals on T2-weighted imaging, and high signal intensity on T2-FLAIR. The lesion exhibited marked enhancement on contrast-enhanced scans, with pronounced adjacent dural enhancement and a distinct dural tail sign. No abnormal hyperintensity was detected on DWI, and the ADC map showed no evident diffusion restriction. Moreover, no abnormal vascular malformations were observed in the intracranial arteries. Preoperative thyroid-related laboratory tests were all within normal ranges, including free triiodothyronine, serum free thyroxine, thyroid-stimulating hormone, anti-thyroid peroxidase antibody, anti-thyroglobulin antibody, and thyroglobulin levels. Thyroid ultrasonography revealed multiple nodules classified as TI-RADS category 3. Based on the patient's imaging findings—well-defined lesion margins, positive enhancement, and a prominent dural tail sign—a preliminary diagnosis of meningioma was considered. Under general anesthesia, she underwent resection of the left temporal mass (Figures 2A,C). Intraoperatively, the tumor was adherent to the overlying muscle and protruded extracranially by 3 × 2 × 2 cm. The capsule was intact. The lesion was markedly hypervascular, red, and had a “fish-flesh” texture; brisk intraoperative bleeding necessitated blood transfusion (Figure 2D Postoperative CT scan).

Postoperative pathology of the left temporal lesion, integrated with immunohistochemistry, supported a diagnosis of metastatic follicular thyroid carcinoma (Figure 2B). The immunophenotype was: cytokeratin (CK) positive, thyroid transcription factor-1 (TTF-1) positive, thyroglobulin (Tg) positive; epithelial membrane antigen (EMA) negative, somatostatin receptor 2 (SSTR2) negative, progesterone receptor (PR) negative, S-100 negative, glial fibrillary acidic protein (GFAP) negative, CD34 negative, calcitonin negative, synaptophysin (Syn) negative; Ki-67 proliferation index 5%.

## Discussion

Follicular thyroid carcinoma (FTC) is the second most common subtype of thyroid cancer. Owing to the gland's rich vascularity, the rate of distant metastasis reaches 15%–27% (10), most frequently to the lungs, lymph nodes, and mediastinum, whereas intracranial spread is rare (10). Moreover, cranial/cranio-cerebral metastases from thyroid carcinoma are typically epidural; direct dural involvement is exceptionally uncommon. Prior series show that most brain metastases originate from lung, breast, and gastrointestinal primaries, with only sporadic cases arising from thyroid carcinoma—underscoring the rarity of intracranial metastasis from this origin. In the present case, the admission presentation and neuroimaging favored meningioma; however, management strategies and prognoses differ markedly among these entities, making careful differential diagnosis—integrating clinical features, imaging, and laboratory evaluation—particularly important. Meningioma is the most frequently reported primary central nervous system (CNS) tumor, accounting for ∼36.1% of all CNS neoplasms with an incidence of 7.61 per 100,000 persons (23). Meningiomas arise predominantly from arachnoid cap cells, and may also derive from dural fibroblasts or pia mater cells—cellular origins that are distinct from those of FTC. Clinically, their expansile growth commonly produces headache and seizures; depending on lesion location, patients may also develop visual decline, visual field deficits, anosmia, hearing loss, or motor dysfunction. Because brain metastases from thyroid carcinoma can also grow in an expansile, mass-like fashion, they may present with similar symptoms. In addition, previous reports have described exophthalmos, unilateral loss of the pupillary light reflex, and necrosis of bone and soft tissue at the lesion site complicated by infection and hemorrhage (4, 8, 11, 12). Nearly all reports note marked tumor hypervascularity, a feature reminiscent of angiomatous meningioma; however, angiomatous meningioma is characterized by abundant vessels and venous sinusoids with a relatively sparse collagenous stroma derived from arachnoid cap cells (13), which can aid in distinguishing it from dural metastasis of thyroid carcinoma. Beyond angiomatous meningioma, multiple dural-based entities can “mimic” meningioma radiologically, including solitary fibrous tumor/hemangiopericytoma (SFT/HPC), lymphoma, and various metastases. Red-flag imaging features that warrant caution include absence of the typical dural tail, adjacent osseous destruction or erosion, strikingly low or high T2 signal intensity, and involvement of the leptomeninges (subarachnoid space); when present, these findings should heighten suspicion for non-meningiomatous pathology (14–16).

Synthesizing prior reports with our index case, we consider gross-total surgical resection the preferred treatment for intracranial (dural) metastases of thyroid carcinoma. The primary goals are to relieve the constellation of symptoms caused by tumor mass effect on normal brain tissue and to prevent further neurological injury. Consistent with the literature, these lesions are highly vascular (4); in our dural-involved case the vascularity was particularly pronounced, and surgery was marked by brisk hemorrhage over a short interval. Without meticulous preoperative preparation, excessive intraoperative blood loss is likely. Regardless of whether complete resection is achieved, patients should undergo comprehensive thyroid carcinoma–directed adjuvant therapy—including radioactive iodine (^131^I) and, as appropriate, radiotherapy and/or systemic therapy—to further improve survival.

Regarding metastatic routes and prognosis, follicular thyroid carcinoma disseminates predominantly via the hematogenous pathway. Intracranial dural involvement generally arises through two mechanisms: (i) direct invasion of the dura from a calvarial metastasis, and (ii) hematogenous seeding of the dura (with a minority potentially via retrograde flow through the vertebral venous plexus). Compared with parenchymal brain metastases, osseous/dural disease is more often solitary, well circumscribed, and amenable to *en bloc* resection. When systemic disease is controlled, integration with stereotactic radiosurgery and systemic therapy confers superior local control and survival, whereas surgery alone seldom yields a durable survival advantage (17, 18).Additionally, the therapeutic landscape for thyroid malignancies continues to evolve, particularly for refractory disease. In a recent study, Fanciulli and colleagues underscored the need to reinvigorate clinical trials in thyroid cancer (e.g., medullary thyroid carcinoma) and examined the promise of novel approaches such as proteasome inhibitors (19). These observations suggest that, as our understanding of tumor biology deepens, proactive clinical investigation will open new avenues for the treatment of thyroid malignancies.

Integrating prior literature, meningeal metastasis from follicular thyroid carcinoma (FTC) generally carries favorable treatment outcomes; most lesions are solitary and manifest as convexity meningioma–like masses. These characteristics substantially reduce operative complexity and enhance surgical efficacy. Across available follow-up, no mortality events were reported. Accordingly, when a new case is suspected to represent meningeal metastasis from FTC, timely surgical management should be pursued to avert progression to more serious sequelae (24). In recent years, radiomics and deep learning have shown promise for the qualitative assessment of dural-based lesions—for example, MRI-based models that distinguish meningioma from its radiographic mimics and that predict biological behavior (20). In the thyroid domain, ultrasound-based AI has likewise been shown to improve benign–malignant discrimination and reduce unnecessary biopsies. Given the extreme rarity of intracranial dissemination from thyroid carcinoma, population-level brain imaging is not warranted; instead, AI is best positioned as a secondary alert and triage tool in high-risk DTC/FTC cohorts—integrating age, histologic subtype, presence of distant metastasis or RAI-refractory disease, serum thyroglobulin trajectories, and neurologic symptoms—to trigger indicated contrast-enhanced MRI and whole-body evaluation (21). Such models should be threshold-calibrated to background incidence and undergo multicenter external validation to mitigate false positives in low-prevalence settings. Current evidence-based guidance likewise advises against screening asymptomatic adults for thyroid cancer (U.S. Preventive Services Task Force, Grade D recommendation) (22).

## Conclusion

Our case and the literature review indicate that intracranial (dural) metastasis from follicular thyroid carcinoma (FTC) is exceedingly rare and typically presents as a solitary convexity dural-based lesion that mimics meningioma and is markedly hypervascular. For operable disease, *en bloc* gross-total resection yields favorable initial control; however, durable benefit generally requires integration with radioactive iodine (^131I) and, when appropriate, stereotactic radiosurgery and/or systemic therapy, as surgery alone seldom confers a long-term survival advantage. Beyond contrast-enhanced MRI, preoperative assessment of intra- and extracranial vascular supply (e.g., MRA/MRV or equivalent angiography) is recommended to mitigate the risk of major intraoperative hemorrhage and to guide surgical planning. No early surgery-related deaths were observed across available follow-up, although recurrence occurred in individual cases; long-term outcomes remain uncertain and warrant larger studies. Given the extreme rarity of intracranial dissemination, population-level brain imaging is unwarranted; in high-risk DTC/FTC cohorts, AI/radiomics tools that integrate clinical and biologic features are better suited as secondary alerts and triage to trigger indicated contrast-enhanced MRI and whole-body evaluation. Future work should include multicenter, prospective studies with standardized reporting and external validation of radiomics/AI models, refinement of risk stratification, and exploration of novel systemic options for refractory disease.

## Data Availability

The original contributions presented in the study are included in the article/Supplementary Material, further inquiries can be directed to the corresponding authors.
