# Correction: Meningeal metastatic tumor with bone destruction from follicular thyroid carcinoma: a case report and literature review

**DOI:** 10.3389/fsurg.2026.1782451

**Published:** 2026-01-22

**Authors:** Wei Liu, Lanming Su, Qinglu Zhang, Yuanqin Liu

**Affiliations:** 1 Department of Neurosurgery, The First Affiliated Hospital of Shandong First Medical University and Shandong Provincial Qianfoshan Hospital, Jinan, China; 2 Department of Neurology, The Second Affiliated Hospital of Shandong First Medical University, Taian, Shandong, China; 3 Department of Ultrasound Medicine, Shandong Provincial Third Hospital, Shandong University, Jinan, Shandong, China

**Keywords:** bone destruction, follicular thyroid carcinoma, intracranial metastasis, meningeal metastasis, meningioma mimicry

Affiliation 2 “Department of Neurology, The Second Affiliated Hospital of Shandong First Medical University, Taian, Shandong, China” was omitted for author Wei Liu. This affiliation has now been added to the author.

Author Qinglu Zhang was erroneously assigned to affiliation 2 “Department of Neurology, The Second Affiliated Hospital of Shandong First Medical University, Taian, Shandong, China”. The author correct affiliation is affiliation 3 “Department of Ultrasound Medicine, Shandong Provincial Third Hospital, Shandong University, Jinan, Shandong, China”.

Author Yuanqin Liu was erroneously assigned to affiliation 3 “Department of Ultrasound Medicine, Shandong Provincial Third Hospital, Shandong University, Jinan, Shandong, China”. The author correct affiliation is affiliation 1 “Department of Neurosurgery, The First Affiliated Hospital of Shandong First Medical University and Shandong Provincial Qianfoshan Hospital, Jinan, China”.

